# EPIGENETIC PROFILES OF ALZHEIMER’S DISEASE

**DOI:** 10.1093/geroni/igz038.3407

**Published:** 2019-11-08

**Authors:** Kyra Thrush, Morgan E Levine

**Affiliations:** 1 Yale University, New Haven, Connecticut, United States; 2 Yale University School of Medicine, New Haven, Connecticut, United States

## Abstract

Although age is highly correlated with incidence of Alzheimer’s Dementia (AD), the field continues to lack a clear understanding of how either normal and/or pathological aging processes drive neurodegeneration. As such, there remains a clear lack of valid and reliable clinical biomarkers to predict that disease’s future development and severity. Epigenetic age based on DNA methylation (DNAm) in brain have been shown to relate to AD neuropathology and cognitive decline. However, they were not initially designed as AD biomarkers. We hypothesized that supervised and unsupervised machine learning techniques (e.g. network analysis, clustering, and regressed-based techniques) could be used to build composite scoring variables from DNAm data that are predictive of AD progression. This work analyzes the methylation of 3 brain regions (cerebellum (CBM), prefrontal cortex (PFC), striatum (ST))—totaling 1,047 brain methylation samples. The samples contain neuropathologically confirmed AD cases and controls, and is enriched for APOE4+ carriers. Detailed subject-level information concerning cognitive measures, lifestyle choices, medications, and neuropathology at death were also considered. Based on epigenome-wide association study (EWAS), we identified a CpG in AIMP2 that is a robust predictor of AD-related phenotypes. Using network analysis, we have also identified co-methylation modules that relate to multifactorial AD phenotypes. Following validation, we intend to follow-up on the biological processes and molecular pathways associated with these epigenetic signatures. In moving forward, predictors of AD diagnosis and prognostication have major implications for early detection and treatment of this major age-related disease.

